# White Matter Lesions and Cognitive Impairment as Silent Cerebral Disease in Hypertension

**DOI:** 10.1100/tsw.2006.99

**Published:** 2006-04-21

**Authors:** Cristina Sierra, Antonio Coca

**Affiliations:** Hypertension Unit, Hospital Clinic of Barcelona, University of Barcelona, Institut d'Investigacions Biomédiques August Pi i Sunyer (IDIBAPS), 170-Villarroel, 08036-Barcelona, Spain

**Keywords:** essential hypertension, cerebral white matter lesions, target organ damage, magnetic resonance imaging

## Abstract

Although the pathogenesis and clinical significance of cerebral white matter lesions remain controversial, it is well established that age and hypertension are the most important factors related to the presence of these lesions. Hypertension is known to be the most important factor for developing stroke and vascular dementia. In addition, the presence of cerebral white matter lesions is an important prognostic factor for the development of stroke, and also for cognitive impairment and dementia. The mechanisms underlying hypertension-related cognitive changes are complex and are not yet fully understood. Correlations between cerebral white matter lesions and elevated blood pressure provide indirect evidence that structural and functional changes in the brain over time may lead to lowered cognitive functioning when blood pressure control is poor or lacking.Some authors have suggested that the presence of white matter lesions in hypertensive patients could be considered an early marker of brain damage.

Cerebral white matter lesions (WML) are frequently seen on brain magnetic resonance imaging (MRI) in healthy elderly people. The pathogenesis of cerebral WML is poorly understood. However, most studies have shown that age, hypertension, diabetes mellitus, and a history of stroke or heart disease are the most important factors related to the presence of WML[1]. Indeed, besides age, hypertension is constantly reported to be the main risk factor for cerebral WML. Pathologic cerebrovascular changes related to hypertension include vascular remodeling, impaired cerebral autoregulation, cerebral microbleeds, WML, lacunar infarcts, and Alzheimer-like changes such as amyloid angiopathy and cerebral atrophy.

The association between WML and hypertension may be mediated through several different pathogenetic pathways, and in this sense, the presence of WML has been reported to be associated with the severity of high blood pressure (BP) values[2], lack of BP control in treated hypertensive patients[3], and nocturnal BP dip[4]. In addition, the relationship between some genetic polymorphisms and the presence of WML in hypertensive patients has been recently reported[5]. The presence of cerebral WML is an important prognostic factor for the development of stroke[6] and also for cognitive impairment[7], and could be considered an early marker of brain damage in essential hypertensive patients.

## PATHOGENESIS OF CEREBRAL WHITE MATTER LESIONS

Various pathologic studies have suggested that cerebral WML are caused by arteriolosclerosis, which can provoke ischemia, and for which hypertension is the main risk factor in addition to the aging process. Indeed, the main hypothesis regarding the association between high BP and WML is that long-standing hypertension causes lipohyalinosis of the media and thickening of the vessel walls, with narrowing of the lumen of the small perforating arteries and arterioles that nourish the deep white matter[1]. The perforating vessels, which originate from cortical and leptomeningeal arteries, have a relatively poor anastomotic system, which makes the white matter vulnerable to cerebral ischemia. In this sense, low BP has also been reported to be a risk factor for WML[1]. Hypertension may also cause disturbances in the blood-brain barrier, which may cause lesions in the white matter by cerebral edema, by activation of astrocytes or by destructive enzymes or other poisons that pass through the damaged vessel walls[1].

Postmortem studies have indicated that WML seen on MRI scans are associated with degenerative changes in arterioles that are related to atherosclerosis, suggesting that cerebral arteriosclerosis of the penetrating vessels is the main factor in the pathogenesis of WML[1]. Bots et al.[8] reported that WML were related to atherosclerosis, indicated by increased common carotid intima-media thickness and carotid plaques. In the same way, de Leeuw et al.[9] showed that the presence of aortic atherosclerosis during midlife, assessed on abdominal radiographs, was significantly associated with the presence of periventricular WML 20 years later.

There are several vascular risk factors that have been related to WML. Moreover, the greater the number of vascular risk factors for cerebrovascular disease, the higher the presence and severity of WML. In the review of Pantoni and Garcia[1], which included more than 160 publications about WML, those studies with multivariate analysis showed that diabetes mellitus and hypertension were associated with WML. However, with regard to lipid abnormalities and smoking status, there are controversial results.

On the other hand, there are studies that have reported that WML are associated with a history of stroke, lacunar infarcts, heart disease, and atrial fibrillation, which are frequently associated with both hypertension and vascular risk factors[1].

## GENETIC FACTORS AND CEREBRAL WHITE MATTER LESONS

Genetic risk factors have been implicated in the presence and severity of WML, but remain undetermined so far. A recent genetic study on elderly twins indicated that the susceptibility to white matter hyperintensity on brain MRI was largely determined by genetic factors[10]. Genes contributing to interindividual variation in BP levels and essential hypertension may play a role in the etiology of WML or stroke, either through their effects on BP levels or through separate pathways. In this sense, the renin-angiotensin system is an example of a system that may be involved in the pathogenesis of both hypertension and arteriosclerosis. Kario et al.[11] found a positive association between angiotensin-converting enzyme D allele and the presence of both silent and clinically overt stroke in Japanese hypertensives. Sierra et al.[5] reported an association between the presence of the DD genotype or the D allele of the angiotensin-converting enzyme gene and WML in asymptomatic middle-aged hypertensive patients.

Among the various genes that potentially predispose to WML in the presence of arterial hypertension, the apolipoprotein E (apoE) gene is one of the best candidates. This is because the apoE, which is encoded by the apoE gene, plays a crucial role in lipid metabolism and neuronal repair after injury of any type. On the other hand, the APOE ε4 allele (one of the three polymorphic forms of the apoE gene) is associated not only with the vascular risk factors for WML[12], but also with its consequences, particularly cognitive impairment and dementia[13]. A recent study confirmed that apoE ε4 carriers with hypertension are most severely affected with respect to cognitive impairment[14].

In this sense, De Leeuw et al.[15] have recently shown, in 971 subjects aged 60–90 years (>50% of them were hypertensives), that the coexistence of an ε4 allele and arterial hypertension is strongly associated with the presence of subcortical WML, while neither hypertension alone nor the presence of an

ε4 allele alone are. This interaction may reflect a decreased capacity for neuronal repair in the presence of one or two ε4 alleles. The results of this study support the hypothesis that the effect of arterial hypertension on the cerebral white matter is enhanced in ε4 carriers.

## CEREBRAL BLOOD FLOW AND WHITE MATTER LESIONS

High BP influences the cerebral circulation causing adaptative vascular changes. Thus, hypertension influences the autoregulation of cerebral blood flow by shifting both lower and upper limits of autoregulatory capacity towards a higher BP, and hypertensive patients may be especially vulnerable to episodes of hypotension[1], which may play a role in the development of WML. An increase in cerebral vascular resistance could be due to narrowing of the small vessels by lipohialynosis and microatherosclerosis. The effect of high BP on small vessels is well known, with vascular "remodeling" occurring in cerebral blood vessels during chronic hypertension. It has been suggested that this structural alteration impairs autoregulation, exposing the white matter to fluctuations in BP. In this sense, a hemodynamic contribution to WML has been found, and it has been hypothesized that changes in cerebral hemodynamics may play a role in the development of WML[16].

However, most studies have found no significant changes in resting cerebral blood flow in both normotensive and hypertensive individuals with silent WML. On the contrary, with relation to vasomotor reactivity (or vasodilatory capacity) and WML, there are controversial results. Kuwabara et al.[17] reported a close relationship between cerebral hemodynamic reserve capacity, measured by positron emission tomography, and the severity of WML in hypertensive patients. Bakker et al.[18] confirmed the association between decreased vasomotor reactivity and WML, measured by means of a transcranial doppler in 73 elderly individuals, 56% of whom were hypertensives. Conversely, Chamorro et al.[19] showed a preserved vasomotor reactivity in 41 patients (71% hypertensives) with silent WML and first-ever lacunar infarction, although they had an increased cerebrovascular tone, measured by transcranial doppler. In a recent study, an association between the presence of silent cerebral WML and increased cerebrovascular tone were found in middle-aged, never-treated, essential hypertensive patients without affecting either cerebral blood flow velocity or the vasodilatory capacity of cerebral vessels[20]. Using exogenous contrast-based perfusion MRI, O´Sullivan et al.[21] have recently shown that elderly hypertensive patients with WML have a significant reduction in cerebral blood flow of normal-appearing white matter compared with hypertensives without WML, suggesting that hypoperfusion may be an early feature in the development of WML. Nevertheless, the question of whether hypoperfusion is a primary pathogenic mechanism or simply a secondary effect of damaged tissue remains unanswered.

## PREVALENCE OF CEREBRAL WHITE MATTER LESIONS

Several studies have examined the prevalence of WML in both normotensive and hypertensive subjects. The ARIC study[3] reported a 24.6% prevalence of WML among individuals aged 55–72 years, 49% of whom were hypertensives. The Cardiovascular Health Study[22] found a prevalence of 33.3% in individuals aged 65 years or older, 44% of whom were hypertensives. The prevalence was 27% in the Rotterdam Study[23], which included individuals aged 65–84 years, 39% of whom were hypertensives. Shimada et al.[2] studied 28 normotensives and 20 hypertensives aged 59–83 years and found a prevalence of advanced WML of 25 and 40%, respectively. The study of Goldstein et al.[24], performed in 144 normotensive individuals aged 55–79 years (10% with casual elevations of BP), showed a 54.9% prevalence of WML. Differences in the prevalence of WML among studies may be due to subtle variations in WML assessment, but especially to the different impact of risk factors such as age and hypertension, which are influenced by the subject criteria selection. In this sense, most studies included both normotensive and hypertensive patients (untreated and treated), or subjects with a wide range of ages or only elderly people. In this sense, in a cohort of 66 untreated hypertensives aged 50–60 years, Sierra et al.[25] reported a prevalence of WML of 40.9%.

## CEREBRAL WHITE MATTER LESIONS, BLOOD PRESSURE, BLOOD PRESSURE VARIABILITY, AND ANTIHYPERTENSIVE THERAPY

The association between hypertension and WML has been established in cross-sectional[1,2,3,22,23] and longitudinal studies[26,27,28,29,30]. However, some reports have suggested that this relationship is only evident when 24-h ambulatory BP monitoring (ABPM) is used for the assessment of BP. In this sense, Goldstein et al.[24] found a correlation between WML and office systolic, but not diastolic, BP in a group of elderly normotensive subjects. Conversely, the severity of WML correlated with both systolic and diastolic BP, measured by ABPM. In a group of mixed normotensives, "white coat" hypertensives, and sustained hypertensives, Shimada et al.[2] also found a correlation between the number of lacunae and periventricular hyperintensities with 24-h BP, but not with office BP.

Concerning circadian pattern of BP, Shimada et al.[4] reported that both nondippers and extreme dippers had significantly more silent cerebrovascular damage (measuring both lacunae and WML) than dippers. Although BP variability has been related to target organ damage in hypertension, its relationship with cerebral alterations has not been established. A previous report by Goldstein et al.[24] suggested a higher standard deviation of awake systolic BP in patients with more severe WML. On the other, neither the circadian rhythm nor the long-term variability of BP were related to WML in a group of 66 middle-aged, never-treated hypertensive patients[25].

Cross-sectional population-based MRI studies have shown that treated and controlled hypertensive patients have less prevalence of WML than untreated hypertensives, and also treated, but not controlled, hypertensive patients[3]. In 1805 individuals from 10 European cohorts, aged 65–75 years, in which BP measurements were initiated 5–20 years before the brain MRI, Van Dijk et al.[27] showed that people with poorly controlled hypertension had a higher risk of severe WML than those without WML, or those with controlled or untreated hypertension. In this study, an increase in systolic and diastolic BP levels were associated with more severe WML, and also a decrease in diastolic BP was associated with more severe periventricular WML. Authors suggest that successful treatment of hypertension may reduce the risk of WML, but a potential negative effect of decreasing diastolic BP level on the occurrence of severe periventricular WML should be taken into account. However, it is important to notice that this lack of difference between controlled hypertensive patients and nontreated hypertensives could be due to the fact that the untreated group had less-severe hypertension or shorter duration of hypertension. Another study performed in 845 subjects with a follow-up of 4 years showed that hypertension at baseline was significantly associated with an increased risk of having severe WML at 4-year follow-up. When taking into account both BP levels and antihypertensive drug intake, analysis showed that the risk of having severe WML was significantly reduced in subjects with normal BP taking antihypertensive medication compared with those with high blood pressure taking antihypertensive drugs[26]. In this study, the brain MRI was performed at the end of the study (at 4-year follow-up).

On the other hand, in a longitudinal study Schmidt et al.[28] evaluated volunteers without neuropsychiatric disease, aged 50–75 years, who underwent brain MRI at baseline and at each follow-up: 3 years (204 individuals) and 6 years (191 individuals). At 3 years of follow-up, results showed that diastolic BP and WML at baseline were the only significant predictors of white matter hyperintensity progression[29]. At 6 years of follow-up, grade of WML at baseline predicted progression of WML better than age and hypertension[28].

Data concerning the impact of antihypertensive treatment on WML are indirect. We have to wait for clinical trials specifically designed for measuring the impact of antihypertensive therapy on WML progression.

## COGNITIVE FUNCTION, HYPERTENSION, AND CEREBRAL WHITE MATTER LESIONS

Hypertension is known to be the most important factor for developing macrovascular cerebral complications such as stroke[31] and, consequently, vascular dementia[31]. Hypertension may also predispose to the development of more subtle cerebral processes based on arteriolar narrowing or microvascular pathological changes. It has been suggested that cerebral microvascular disease contributes to the development of vascular cognitive impairment[32]. Results from cross-sectional[33] and longitudinal[34,35,36,37] studies have shown a correlation between BP and cognitive function in elderly people. These studies have reported an association between high systolic BP (SBP) (the Honolulu-Asia Aging Study[35]), high diastolic BP (DBP) (the Uppsala Study[36]), elevated SBP and DBP (the Framingham Study[34]), or hypertension (National Heart, Lung, and Blood Institute Twin Study[37]) at midlife and impaired cognitive performance in late life. In addition, there is some evidence that antihypertensive drug treatment could play a role in the prevention of cognitive impairment[38] or vascular dementia[39] through BP control.

On the other hand, the presence of cerebral WML is an important prognostic factor for the development of stroke[40,41] or stroke recurrence[6,42,43], and also for cognitive impairment[7,22,23] and dementia[44].

Various studies have shown an association between the presence of cerebral WML and cognitive function in both normotensive and hypertensive elderly populations[7,22,45,46,47]. In a longitudinal study, de Groot et al.[7] examined the relation between severity of WML and cognitive decline over a 10-year period in 563 elderly subjects (60–90 years), and they found that subjects with severe periventricular WML had more rapid cognitive decline. An association between the presence of WML in brain MRI and the existence of poorer performance on neuropsychological tests were found in middle-aged, asymptomatic, never-treated, essential hypertensive patients[48]. In this study, hypertensive patients with WML had a significantly poorer digit span forward performance, a standardized measure of attention, and slightly lower scores on visual memory test than hypertensives without WML[49]. In addition, it has been reported in a longitudinal study performed in 1077 people aged 60–90 years who underwent a brain MRI at baseline and were followed-up for a mean of 5.2 years, that WML especially in the periventricular region increase the risk of dementia (76%: Alzheimer´s disease; 13%: vascular dementia; 11%: other types of dementia)[44]. In the same way, in the Cardiovascular Health Study, individuals with more severe WML have a twofold increased risk of dementia[49]. In this study, 3608 participants that had a brain MRI at baseline in 1991 were followed to 1998–1999. There were 480 incident dementia cases, 330 (69%) were classified as Alzheimer's disease. In this study, apolipoprotein E $\varepsilon 4$ genotype was also a powerful predictor of dementia.

The mechanisms underlying hypertension-related cognitive changes are complex and are not yet fully understood. It is unclear whether the impact of elevated BP on cognitive decline in late-life is mediated through its chronic and negative effect on the structural characteristics of the brain. Correlations between cerebral WML and elevated BP provide indirect evidence that structural and functional changes in brain over time may lead to lowered of cognitive functioning when BP control is poor or lacking. Skoog et al.[50] reported an association between elevated BP at age 70 and the development of dementia 10–15 years later, while patients with WML at age 85 had a higher BP at age 70, suggesting that previously increased BP may increase the risk of dementia by inducing small-vessel disease and WML. In the same way, Swan et al.[51] showed that midlife SBP is a significant predictor of WML and decline in cognitive function.

In summary, there are several relationships that connect hypertension with WML, stroke, cognitive impairment, and dementia, and also different relationship among them (Fig. 1). However, the mechanism that would explain all these relationships remains to be fully explored.

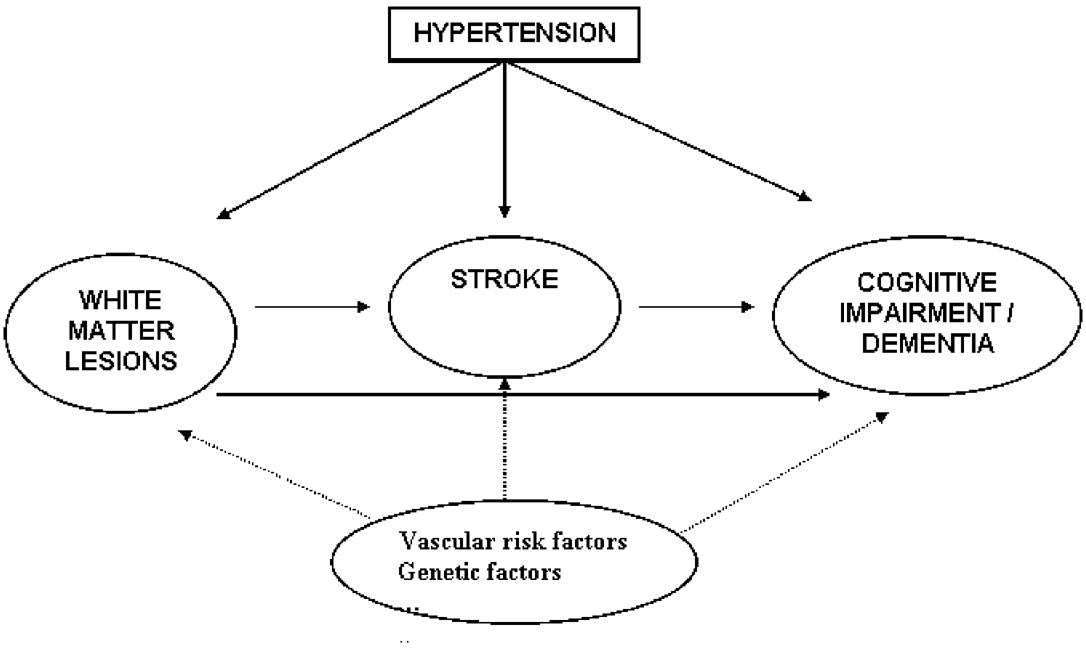

**FIGURE 1**. Different connections among hypertension, WML, and cognitive impairment.

## CONCLUSION

Several pathologic studies have suggested that cerebral WML are caused by arteriolosclerosis, for which hypertension, besides aging, is the main risk factor. Indeed, hypertensive patients have higher rate and extent of areas of WML compared with controls, and also with treated and controlled hypertensives. On the other hand, it is known that hypertension is the main risk factor for stroke and vascular dementia. Similarly, the presence of cerebral WML is an important prognostic factor for the development of stroke and also for cognitive impairment, and dementia.

Hypertension is associated with silent organ damage that precedes the development of clinical events related to cardiovascular disease. Like left ventricular hypertrophy in the heart, microalbuminuria in the kidney or endothelial dysfunction and intima-media thickness in the vascular wall, WML could be considered the early process of brain damage in hypertension.

The presence of WML may play a role in the development of cognitive impairment in essential hypertension. It is established that the prevalence and incidence of vascular dementia increase exponentially after 70 years of age, but little is known about the cognitive status of middle-aged individuals. Cognitive decline is, perhaps, not an inevitable consequence of aging. In fact, hypertension appears to predispose patients to the development of cognitive impairment and dementia, after a period varying from a few years to several decades, in which subtle cognitive impairment could already be detected, and related to the presence of early cerebrovascular damage, such as WML. Nevertheless, exactly how early high BP may begin to exert their influence is not well understood.
